# Adsorption of Copper (II) from Aqueous Solutions with Alginate/Clay Hybrid Materials

**DOI:** 10.3390/ma14237187

**Published:** 2021-11-25

**Authors:** Maria Râpă, Anca Andreea Ţurcanu, Ecaterina Matei, Andra Mihaela Predescu, Mircea Cristian Pantilimon, George Coman, Cristian Predescu

**Affiliations:** 1 Faculty of Materials Sciences and Engineering, University Politehnica of Bucharest, 313 Spl. Independentei, 060042 Bucharest, Romania; maria.rapa@upb.ro (M.R.); ecaterina.matei@upb.ro (E.M.); andra.predescu@upb.ro (A.M.P.); mircea.pantilimon@upb.ro (M.C.P.); george.coman@upb.ro (G.C.); 2 Center for Research and Eco-Metallurgical Expertise, Faculty of Materials Science and Engineering, University Politehnica of Bucharest, 313 Spl. Independentei, 060042 Bucharest, Romania

**Keywords:** activated clay, alginate, copper removal, size distribution, absorption kinetic, absorption isotherm

## Abstract

Massive amounts of industrial and agricultural water around the world are polluted by various types of contaminants that harm the environment and affect human health. Alginic acid is a very versatile green polymer used for heavy metal adsorption due to its availability, biocompatibility, low cost, and non-toxic characteristics. The aim of this paper was to prepare new low-cost hybrid composite beads using sodium alginate with treated montmorillonite and kaolin for the adsorption of copper (Cu) cations. Modified and unmodified clays were investigated by studying their morphology and elemental composition, functional groups, and mean particle size and particle size distribution. The characterization of alginate/clay hybrid composite beads was carried out by evaluating surface morphology (by scanning electron microscopy, SEM), crystallinity (by X-ray diffraction, XRD), and point of zero charge (pH_pzc_)(Zeta Potential Analyzer). Batch adsorption experiments of alginate/clay hybrid composite beads investigated the effect of metal concentration in the range of 1–4 mg L^−1^ on Cu(II) removal, adsorption kinetic for maximum 240 min, and Langmuir and Freundlich adsorption isotherms by using atomic absorption spectrometry. The pseudo-second-order kinetic model best fitted the adsorption for alginate/montmorillonite beads (R^2^ = 0.994), while the diffusion process was predominant for montmorillonite/kaolin beads (R^2^ = 0.985). The alginate/clay hybrid materials best fitted the Langmuir isotherm model.

## 1. Introduction

Massive amounts of industrial and agricultural water around the world are polluted by various types of contaminants that harm the environment and affect human health. Removing contaminants from effluents with sustainable adsorbent materials made from abundant and inexpensive cellulose, alginate, starch, chitosan is a feasible approach to deal with this problem [1,2,3,4,5,6,7]. These polysaccharides are advantageous for the adsorption of heavy metals from aqueous solutions due to their low cost, biodegradability, and reduced carbon footprint and environmental impact.

Among these molecules, alginic acid is a very versatile green polymer used for heavy metal adsorption due to its availability, biocompatibility, low cost, and non-toxic characteristics. Alginates are anionic polysaccharides obtained from seaweed, consisting of a linear chain of (1–4) β-D-mannuronic acid (M) and residues of α-L-guluronic acid (G) arranged in irregular blocks, having applications in interdisciplinary fields such as medicine [8], drug delivery [9], food [10], or heavy metal ion removal [11]. Bacterial alginate was investigated for copper removal [12]. Data showed that 1 g of alginate can immobilize 1.90 mmol L^−1^ of copper. Other authors reported that a solution with a concentration of 1 g L^−1^ acidified alginic acid was able to sequester 54.11% of Cu(II) [5]. Unfortunately, alginic acid in the adsorption process has not an adequate mechanical stability, and usually crosslinking and reinforcement with inorganic materials are applied. Binary composite beads based on alginate and cellulose [13] or starch and nanoclay [14] and ternary green composites containing sodium alginate and chitosan and glass bubble [4] are examples of environmentally friendly adsorbents fabricated to remove contaminants from aqueous solutions.

Clay is a mixture of aluminosilicates that contains silicon, aluminum, manganese, titanium, potassium, calcium, and sodium [15,16,17]. This lamellar structure, with low cost and worldwide distribution, imparts to clays a great ability to adsorb contaminants, helping with their removal [18]. Montmorillonite and kaolin, the most representative of clays, are abundant in nature, low cost, not toxic, water-absorbing and possess an excellent chemical reactivity with respect to an alginate matrix [19,20,21]. Sodium montmorillonite (MTM) is composed of one aluminum octahedral layer between two silicate tetrahedral layers [22]. Kaolin generally consists of aluminum silicate with the addition of magnesium and calcium silicate [23,24]. The lamellar structure forms large specific surfaces and provides the possibility of adsorption of inorganic ions and organic molecules. However, the heavy metal adsorption capability of kaolin is low due to its low cation exchange capacity (15–75 mmol/kg) [17,21,25]. To avoid the separation of clay in the adsorption process, it is incorporated as a filler into alginate/starch compositions to remove the copper cation [14] or cellulose hydrogels [22]. There are known hybrid clay/alginate composites for the removal of copper and 4-nitrophenol pollutant from water [26], total organic acid (TOA) anions as heat-stable salts (HSS) and heavy metal ions (chromium and iron) from the methyl diethanolamine (MDEA) solvent [27] and paint [28], and pentachlorophenol [18] and 2,4-dichlorophenol [29] from aqueous media. The improvement of the affinity of clays for various contaminants is achieved by the addition of chemicals such as sulfuric acid [30] and cationic surfactants [31] or by physical treatments. The acid treatment of clay is a preferred method as it increases the surface contact area, maintaining the silanol groups by augmenting the number of Brönsted sites. It was found that acid-activated natural red clay from Tunisia reached a maximum adsorption capacity of 23.59 mg g^−1^ of Cd ions [18]. Besides for the removal of contaminants, the acid treatment of clays was employed for the decolorization of vegetable, animal, and mineral oils [30] and for the preparation of bio-based lubricants [32]. In this context, our purpose was to develop new low-cost adsorbents based on clay powders and Na alginate, as a renewable and biodegradable biopolymer, for the adsorption of pollutants present in water.

Copper (Cu) is a heavy metal occurring in geological deposits, sites of volcanic activity, and following weathering and erosion of rocks and soils [33]. Anthropogenic sources of copper include mining activities, agriculture, metal and electrical manufacturing [34], as well as pesticides and building industry. A considerable source of copper is represented by drinking water due to Cu leaching from plumbing [34]. Copper concentrations that exceed 1 ppb can be toxic for human and aquatic organisms and ecosystems [35]. The copper concentration in electroless plating wastewater must not exceed 0.5 ppb [36]. According to the EPA, the maximum level of copper ions in drinking water should not exceed 1.3 mg L^−1^ [37]. However, Cu is essential for human metabolism, being responsible for the creation of hemoglobin and hemocyanin and oxygen-managing pigments in the blood of vertebrates and shellfish, respectively. At a concentration ranging from 1 to 10 μg L^−1^, most of the aquatic species are sensitive to the Cu(II) [38]. This highlights the need for novel materials for the removal of low levels of Cu. Precipitation [39,40], chelation [41], and electrodeposition methods [42] are successful for Cu removal from wastewater.

The objectives of this study were: (i) to improve by chemical treatment the affinity of montmorillonite and kaolin for copper cations; (ii) to reinforce sodium alginate with modified clays, thus obtaining new hybrid composite materials; (iii) to use the new hybrid composite materials as potential adsorbents for removing Cu^2+^ ions from synthetic waters by batch adsorption. We hypothesized that the carboxyl (–COO–) and hydroxyl (–OH) functional groups present in alginate and the reactive –OH groups present in mineral clays would act as adsorption sites, facilitating the successful removal of Cu^2+^ from synthetic water.

## 2. Materials and Methods

### 2.1. Materials

Sodium alginate powder (BioChemica, Billingham, UK), (C_6_H_7_O_6_Na)_n_, 90.8% purity, characterized by a molecular mass in the range of 10,000–600,000 g/mol, with max. Pb content of 0.002%, mass loss at drying of max. 15%, and viscosity (1% at 20 °C) of 350–550 mPa·s was used as a polymeric matrix. We prepared a 1% (*w/v*) sodium alginate solution by dissolving sodium alginate into 100 mL of distilled water at a temperature of 90 °C, stirring at 600 rpm for 2 h.

**Figure 1:**
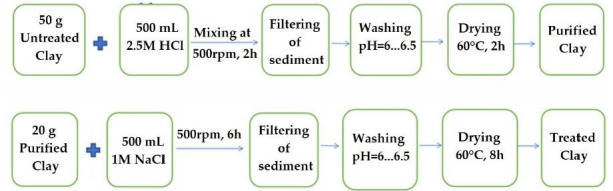
Flow chart showing the chemical treatments of montmorillonite and kaolin, respectively.

Montmorillonite and kaolin clays were acquired from Siceram S.A. (Sighisoara, Romania). In order to increase the cationic exchange capacity, the starting clays were modified according to procedure described by Taleb et al. [43] with little modifications. Shortly, 50 g of each clay type (montmorillonite and kaolin) were mixed with 500 mL of 2.5 M HCl at room temperature for 2 h, stirring at a rate of 500 rpm. Afterwards, the mixture was filtered and washed several times using distilled water and dried in an oven at a temperature of 90 °C for 6 h. Then, 20 g from the dried clay was put into 500 mL of 1 M NaCl solution and stirred at 500 rpm for 6 h at room temperature. The suspension was filtered and washed several times with distilled water to remove the Cl^−^ ions (tested with 0.1 M AgNO_3_ solution). Finally, the samples were dried in an oven at a temperature of 90 °C for 6 h and ground before use. A schematic flow chart illustrating the steps for the chemical modification of clays is shown in Figure 1. Calcium chloride (CaCl_2_ × 2H_2_O), analytical-grade, was used as the crosslinking agent.

### 2.2. Preparation of Calcium Alginate/Clay Hybrid Composite Beads

Modified montmorillonite and kaolin, respectively, 2% *w/v*, were added into the calcium alginate solution under magnetic agitation at a temperature of 90 °C, stirring at 600 rpm for 1 h. In order to eliminate the air bubbles, the obtained solution was sonicated in an ultrasonic bath for 30 min. Calcium alginate/montmorillonite and calcium alginate/kaolin hybrid composite beads were obtained by pouring the previously obtained solutions dropwise into a 1 M CaCl_2_ solution under stirring at 200 rpm, by means of a peristaltic pump. The obtained beads were collected and washed with distilled water and dried in an oven at a temperature of 30 °C for 72 h. Calcium alginate microbeads were prepared in the same conditions, as a reference material. Weight reductions about 7 times and 10 times for alginate/clays beads and calcium alginate beads, respectively, compared with the wet beads, were recorded after drying.

### 2.3. Structural Characterization 

Modified and unmodified clays were investigated by studying their morphology and elemental composition, functional groups, and particle size distribution.

The characterization of alginate/clay hybrid composite beads was carried out by evaluating their surface morphology, chemical structure, mean size and particle size distribution, and crystallinity. 

A scanning electron microscopy (SEM) with field emission using a QUANTA 450 FEG model (FEI, Eindhoven, The Netherlands) coupled with an energy dispersive X-Ray analysis detector (EDX) and a gun at a 1.2 nm resolution, with a resolution of 133 eV was used to characterize the morphology of the materials. Microscopy analysis was performed in low-vacuum mode with a vacuum of 50 Pa. Attenuated total reflectance–Fourier transform infrared spectroscopy (ATR–FT-IR) was performed with an Interspec 200-X Spectrophotometer (Interspectrum, Tartumaa, Estonia) in the spectral region of 4000–750 cm^−1^. Twenty scans were collected for each sample before and after Cu ions adsorption at a spectral resolution of 2 cm^−1^. Point of zero charge (pH_pzc_) measurements for clays particles in suspension were carried out by help of a Zetasizer Nano (Malvern Instruments, Malvern, UK), at the pH values ranging from 2 to 13 adjusted with 1 mM NaCl or 1 mM HCl. The pH_pzc_ value was determined from a plot of initial pH versus the pH of the samples. X-ray diffraction (XRD) analysis was performed using a PANalytical X’Pert PRO MPD spectrometer (Almelo, The Netherlands) with a Cu anode.

### 2.4. Batch Adsorption Experiments

The batch adsorption experiments of alginate/clay hybrid composite beads were performed to evaluate the Cu(II)removal, adsorption kinetics for maximum 240 min, and Langmuir and Freundlich adsorption isotherms with the help of ContrAA 800 atomic absorption spectrometer (Analytik Jena, Jena, Germany) with the flame technique, using solutions with metal concentrations in the range of 1–4 mg L^−1^. A solution of copper in HNO_3_ at a concentration of 1000 mg L^−1^, the ICP multielement standard solution IV (Merck, KGaA, Darmstadt, Germany), was used as the calibration standard.

Cu(II) adsorption and the equilibrium adsorption capacity were calculated using Equations (1) and (2).

(1) $$\mathrm{Removal}\;\mathrm{Efficiency}\;(\%)=\frac{\left(C_{0}-C_{e}\right)}{C_{0}}\times 100$$

where *C*_0_ is the initial adsorbate concentration (mg L^−1^)and *Ce* is the adsorbate concentration (mg L^−1^) at the liquid–phase equilibrium.

The equilibrium adsorption capacity of the hybrid composite beads (*q_e_*) was calculated using Equation (2):

(2) $$q_{e}\left(mg\times g^{-1}\right)=\frac{\left(C_{0}-C_{e}\right)\times V}{m}$$

where *V* represents the volume of adsorbate (L), and *m* is the mass of the adsorbent used (g).

## 3. Results and Discussion

### 3.1. Characterization of Modified Clays

**Figure 2:**
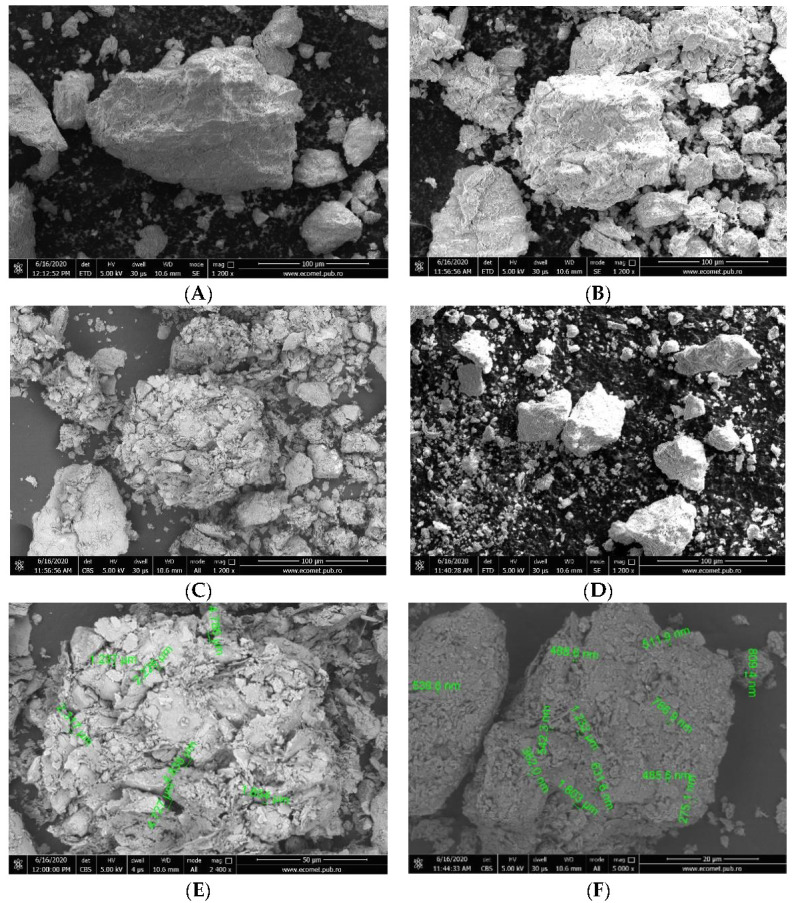
Scanning optical microscope (SEM) images of (**A**) untreated montmorillonite 1200×; (**B**) untreated kaolin 1200×; (**C**) treated montmorillonite 1200×; (**D**) treated kaolin 1200×; (**E**) treated montmorillonite 2400×; (**F**) treated kaolin 5000×. EDS detector with Silicon Drift (SDD) technology, EDAX Octane Plus.

The surface morphology of the clays before and after treatment with HCl and NaCl was studied by SEM analysis and is illustrated in Figure 2A–F.

From the images presented in Figure 2A,B, compact aggregates of clumped particles in the µm size , with lamellar surfaces are visible; kaolin showed surfaces smaller than those of montmorillonite. A similar structure with aggregates was observed on the surface of hydrous aluminum silicate clay mineraloid, an adsorbent used for the removal of Ba(II) [44]. After chemical treatment, the fragmented clays particles with interlamellar spacing and reduced surfaces (smaller surfaces in the case of kaolin) could be seen (Figure 2C,D). The measuring of pore size at the surface revealed a wide size distribution between 1237 µm and 4835 µm in the case of treated montmorillonite (Figure 2E) and between 362 µm and 1803 µm in the case of treated kaolin (Figure 2F). The lamellar structure and the presence of pores in the treated clays formed large specific surfaces, making ion adsorption possible.

**Figure 3:**
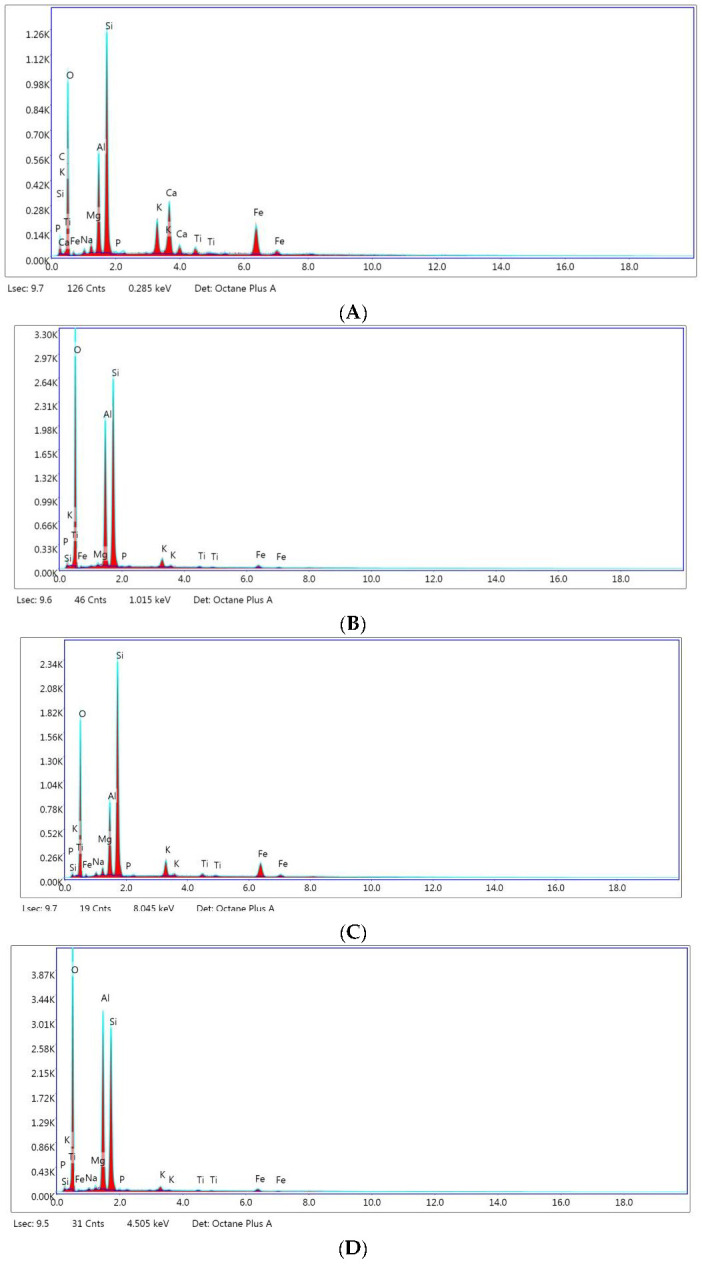
EDS patterns for (**A**) untreated montmorillonite; (**B**) untreated kaolin; (**C**) treated montmorillonite; (**D**) treated kaolin.

Figure 3A–D shows the intensity of chemical elements in treated montmorillonite and kaolin evaluated by X-Ray energy-dispersive spectrometry (EDS).

Table 1 shows the elemental composition of untreated and treated clays.

Data in Table 1 show the presence of Si, Al, Mg, K, Ti, and Fe chemical elements in the structure of the two types of clays. These clays consist of two tetrahedral [MO_4_]^4−^ layers in which M is Si^4+^, Al^3+^, or Fe^3+^ interconnected by an octahedral layer in which the main cations are Al^3+^, Fe^3+^, Mg^2+^, and Fe^2+^. Na is found in the structure of the treated clays (1.19% in the case of montmorillonite and 0.4% in the case of kaolin).

**Figure 4:**
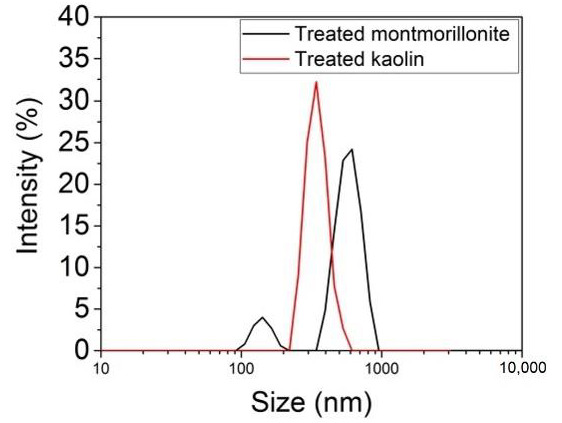
Size distribution of the treated clays by intensity. Test conditions: temperature 25 °C, dispersant solution 1 mM NaCl, equilibrium time 120 s.

The size distribution of the clay particles versus the relative intensity of light scattered by the particles is shown in Figure 4, and the intensity-weighted mean diameter (Z-Average) and polydispersity index (PdI), as a measure of the broadness of the size distribution and the size of the peaks, are presented in Table 2.

As shown in Table 2, the kaolin particles had smaller size than the montmorillonite particles, and all samples were polydispersed. These characteristics, correlated with the morphology determined by SEM, should be beneficial for the adsorption of pollutants from water.

**Figure 5:**
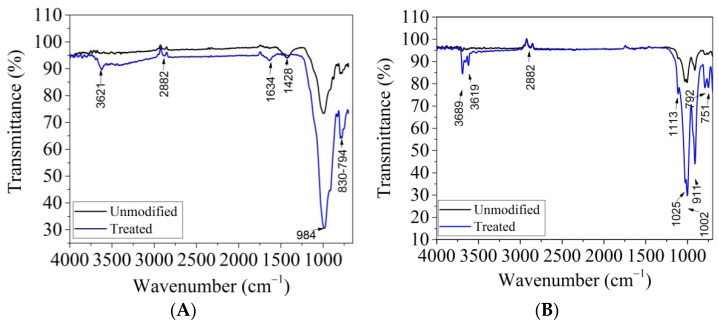
FT-IR spectra of (**A**) montmorillonite and (**B**) kaolin, modified and unmodified.

The FTIR spectra of the original and treated clays are shown in Figure 5.

All spectra exhibited a main band located at 1035 cm^−1^, attributed to the Si–O stretching of montmorillonite. The band located about 915 cm^−1^ was ascribed to the presence of Al_2_OH species that are characteristic of dioctahedral smectites [36], while the band located at 790 cm^−1^ was attributed to the Si_2_OH bending mode. The band located at 840 cm^−1^ was assigned to the AlMgOH bending mode. All absorption spectra shown in Figure 4 show a main absorption band located at approx. 1020 cm^−1^, attributed to the tensile vibration of the Si–O bond. The absorption peak at 910 cm^−1^ in the kaolin structure was due to the presence of Al_2_OH, while the absorption band at 788 cm^−1^ occurred due to the bending vibration of the Si_2_OH group. The absorption spectrum of kaolin after treatment showed two localized peaks at 3689 cm^−1^ and 3619 cm^−1^ attributed to the stretching vibration of Al(OH)Al [32]. This contribution slightly increased after the acid treatment, which seems to indicate the formation of a small proportion of amorphous silica. The absorption band located at approx. 1640 cm^−1^ in the case of montmorillonite was attributed to the bending vibration of the H–O–H group. After treating the clays, the absorption bands of the functional groups increased in intensity.

The characterization of clays performed by SEM/EDAX and FT-IR analyses revealed that the treatment of clays with HCl and NaCl improved the surface properties, allowing the clays to retain heavy metal pollutants.

### 3.2. Characterization of Alginate/Clay Hybrid Composite Beads

**Figure 6:**
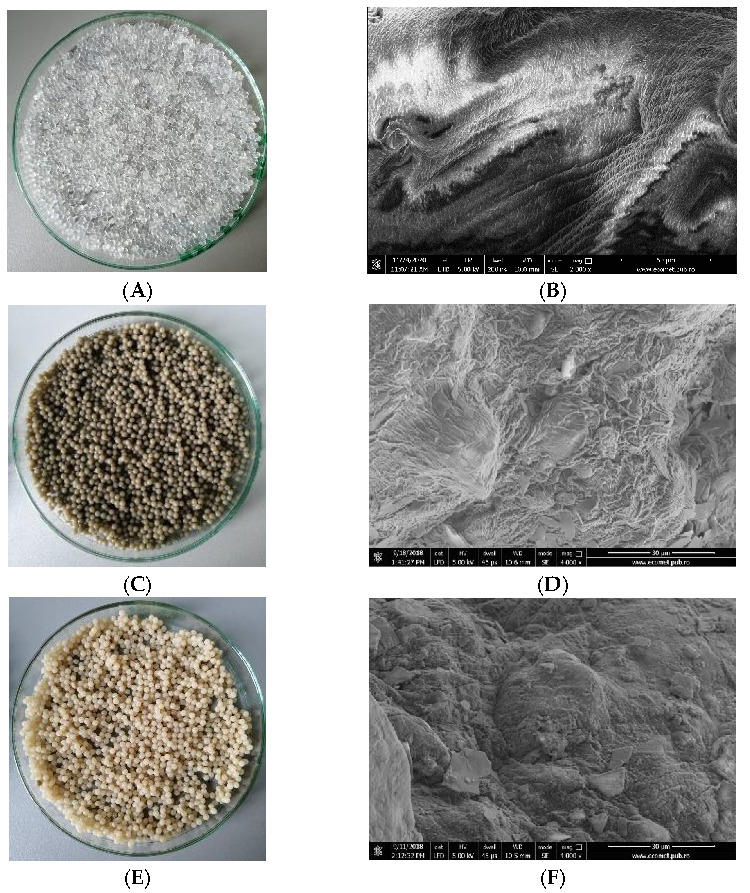
Digital images and SEM micrographs of (**A**,**B**) Ca alginate beads, (**C**,**D**) Ca alginate/montmorillonite beads; (**E**,**F**) Ca alginate/kaolin beads.

The surface morphology of alginate/clay composite beads was studied by SEM analysis, which is illustrated in Figure 6A–F.

SEM analysis revealed that the Ca alginate beads had a smoot surface, while the alginate loaded with montmorillonite/kaolin beads presented irregularities that allow more effective contact with the environment to which they are exposed due to their specific surface (Figure 6). This led to the improvement of the adsorption capacity of the material. The beads displayed a wilted form, which was due to the drying process and the internal vacuum of the SEM equipment, which removed moisture from the samples. It is clearly observable that, although submitted to severe dehydration, the integrity of the samples was maintained, without any cracks forming on the samples’ surface. This is a good indicator of the stability of the materials in the environment, even when subjected to rapidly varying conditions.

**Figure 7:**
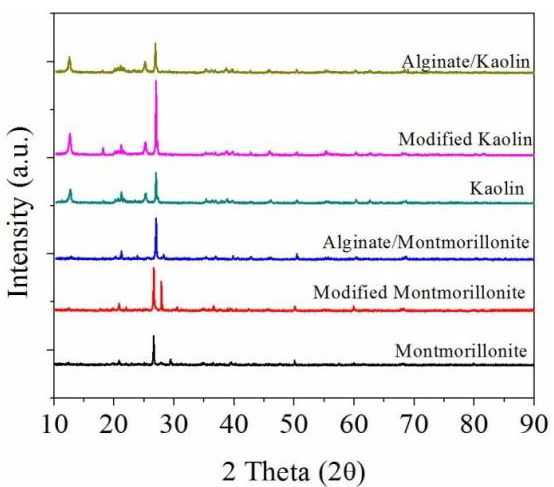
XRD patterns of the investigated alginate/clay as compared with those of montmorillonite and kaolin, before and after modification.

The crystallinity of the prepared beads in comparison with that of the original and treated clays is presented in Figure 7.

The XRD analysis of the samples showed differences in peak intensities for each processing step which the materials underwent. In the case of montmorillonite, the specific peaks for SiO_2_ and sodium calcium aluminum silicate, which are the main components of the base material, could be observed at their specific 2θ angles and Miller indices of 26.62 ((0 1 1) for SiO_2_ and (−1 1 4) for the aluminosilicate); 50.1 ((1 1 2) for SiO_2_ and (0 −4 6) for the aluminosilicate), and 20.84 ((1 0 0) for SiO_2_). Kaolin showed specific peaks for SiO_2_ at 26.62 (0 1 1), 20.84 (1 0 0), and 50.1 (1 1 2), for Al_2_Na_2_O_6_Si at 27.77 (0 4 0), 22.01 (2 0 −2), and 28.07 (0 0 −4), and for TiO_2_ at 24.95 (1 0 1), 47.17 (2 0 0), and 38.01 (0 0 4). By comparing the results in visible peak intensity between the various stages of processing that the base material went through, it is clearly visible that by modifying montmorillonite and kaolin, a more refined structure emerged that displayed clearer crystallinity, which can be associated with a more homogeneous structure of the material.

The addition of alginate, however, reduced the peak intensity due to its organic nature, as it covered the base materials and diluted the X-ray intensity during the analysis. This is also an indicator that the two materials were combined efficiently, which increased the homogeneity of the final product.

**Figure 8:**
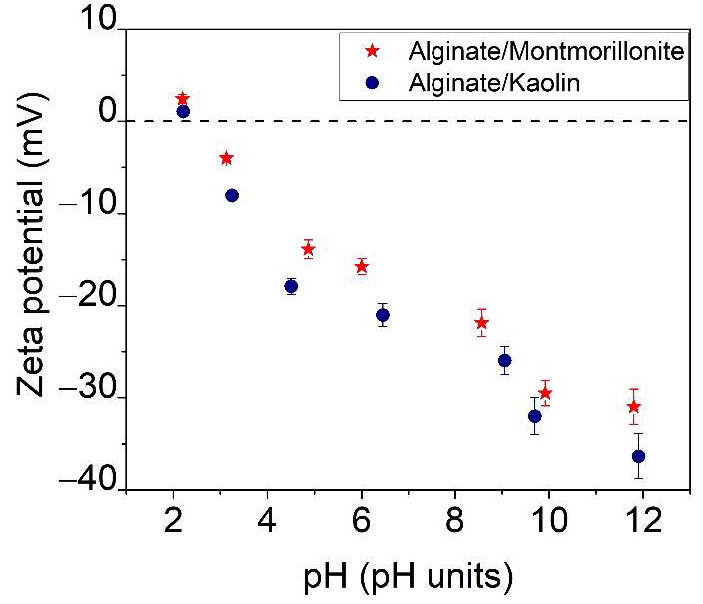
Point of zero charge (pH_pzc_) for the investigated alginate/clays.

Figure 8 shows the alginate/clay particles zeta potential dispersion as a function of pH in range between 2 and 12.

The alginate/montmorillonite and alginate/kaolin particles found in suspension in an NaCl solution exhibited almost similar zeta potentials and point of zero charge at pH 2.7 ± 0.01 for the alginate/montmorillonite beads and 2.4 ± 0.01 for the alginate/kaolin beads. At pH values greater than the pH_pzc_ value, the surface of the hybrid composites became negative, which facilitated the adsorption of positive ions [45]. The adsorption experiments were performed at a pH in the range of 3–4 in order to avoid the protonation/deprotonation of the surface functional ≡Al–OH and ≡Si–OH groups of the clays [44]. In Figure 8, it can be observed that the prepared adsorbents were stable.

The effect of Cu(II) concentration on time was assessed at 1 mg L^−1^, 2 mg L^−1^, 3 mg L^−1^, and 4 mg L^−1^ pollutant concentrations using an amount of 250 mg of adsorbent.

**Figure 9:**
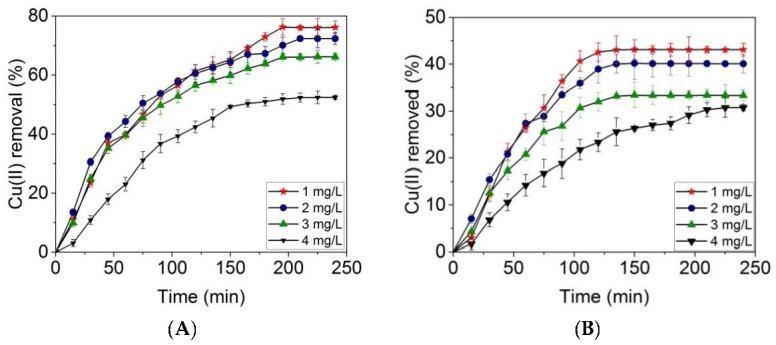
Adsorption tests for alginate/montmorillonite beads (**A**) and alginate/kaolin beads (**B**) investigated at four concentrations of pollutant (1–4 mg L^−1^), using 50 mL adsorbate, with a maximum contact time 240 min and 250 mg of adsorbent.

Figure 9 shows that, by increasing the pollutant concentration from 1 mg L^−1^ to 4 mg L^−1^, the percentage of removed Cu(II) decreased from 76.1 ± 1.6% to 52.4 ± 0.7% in the case of the alginate/montmorillonite beads (Figure 9A) and from 43,1 ± 0,4% to 30.3 ± 1.5% in the case of the alginate/kaolin beads (Figure 9B). This behavior can be explained by the decreasing number of available adsorption sites as the concentration of adsorbate increased. The adsorbable sites became occupied by Cu(II). A similar percent efficiency was reported for ionic liquids that extracted 65% Cu(II) after 60 min from aqueous solutions [46]. In another study, an innovative adsorbent based on cow bones, coconut shells, and zeolite showed 69% and 79% adsorption capacity for Cd(II) and Pb(II), respectively, in the following experimental conditions: 24 h of contact, particle size of 1 mm, and concentration of adsorbent of 12.5 g L^−1^ [45]. As it is also observed in Figure 9, the alginate/montmorillonite beads allowed a significant removal of Cu(II) from the aqueous solution, being more effective than the alginate/kaolin beads. The adsorption capacity at equilibrium of Cu(II) was recorded with a contact time of 210 min for the alginate/montmorillonite beads and of 135 min for the alginate/kaolin beads. The decrease of Cu(II) adsorption in the case of the alginate/kaolin beads as compared with the alginate/montmorillonite beads can be explained by the limited access of Cu(II) ions to the free binding sites, maybe due to repulsive forces between adsorbate and adsorbent.

The dynamic of the adsorption process was investigated by using pseudo-first-order, pseudo-second-order, and Weber’s intraparticle diffusion kinetic models, according to the Equations (3)–(5):

Log(*q_e_* − *q_t_*) = log(*q_e_*) − *k*_1_*t*/2.303 (3)

(4) $$\frac{t}{q_{t}}=\frac{1}{K_{2\;}q_{e}^{2}}+\frac{t}{q_{e}}$$

(5) $$q_{t}=K_{i}t^{0.5}+C_{i}$$

where *q_e_* and *q_t_* are the adsorption capacities at equilibrium and time *t* (mg g^−1^), and *k*_1_, *K*_2_, and *K_i_* are the rate of pseudo-first-order adsorption (l min^−1^), pseudo-second-order adsorption (g/mg min), and intradiffusion, respectively; *C_i_* is an arbitrary constant representing the boundary layer thickness.

The pseudo-first-order kinetic model is based on the hypothesis that the rate of change of solute uptake with time is directly proportional to the difference in saturation concentration and the amount of solid uptake with time. The pseudo-second-order kinetic model states that the rate-limiting step is the chemical sorption or chemisorption and predicts the behavior over the whole range of adsorption. The intraparticle diffusion model assumes that the diffusion of the adsorbate controls the adsorption process.

**Figure 10:**
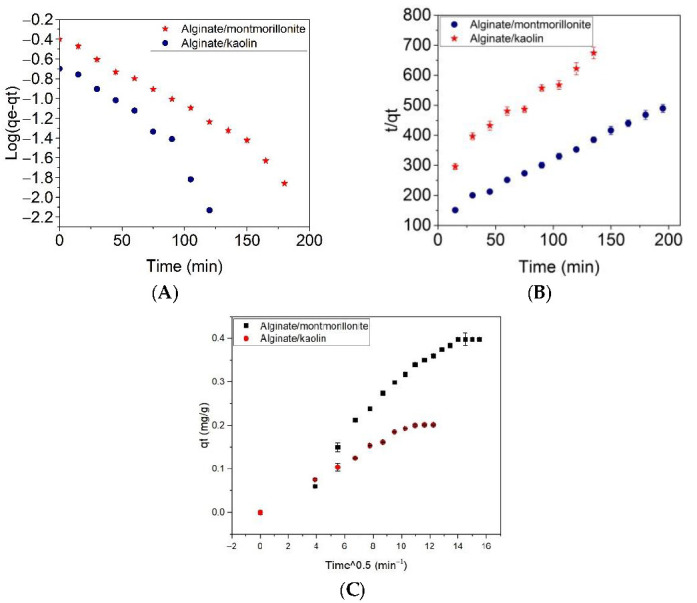
Adsorption kinetic for alginate/clay beads: pseudo first-kinetic model (**A**), pseudo second-kinetic model (**B**), and intraparticle diffusion models; (**C**) pollutant concentration was 3 mg L^−1^.

A plot of Log(*q_e_–q_t_*) versus contact time, *t/q_t_* versus contact time, and adsorbate uptake versus the square root of time (*t*^0.5^) calculated according to Equations (3)–(5), respectively, are shown in Figure 10A–C, and some calculated parameters are listed in Table 3. The rate constants (K) and *q_e_* (mg/g) were evaluated from the slope and intercept of the regression lines.

The adsorption kinetic for alginate/clay beads is shown in Figure 10, and the Lagergren pseudo-first order and pseudo-second order adsorption kinetic models are shown in Table 3.

The results in Table 3 indicated that the pseudo-second-order kinetic model best fitted the adsorption of alginate/montmorillonite (R^2^ = 0.994). A diffusion process was predominant for montmorillonite/kaolin beads (R^2^ = 0.985). It was also observed that the rate constant for montmorillonite beads was in the order diffusion > pseudo-second-order model > pseudo-first-order model. In the case of the intraparticle diffusion model, the adsorbate faster diffused through the solution into the pores of alginate/montmorillonite beads (Ki = 0.027 ± 0.0014 mg/g min^0.5^). This behavior was correlated with the pore dimensions of montmorillonite powder (Figure 2). For the alginate/kaolin beads, the adsorption rate was in the order pseudo-second-order model > pseudo-first-order model > diffusion model. However, the high value of the regression coefficient R^2^ suggested the applicability of intraparticle diffusion and the pseudo-second-order model to the kinetic data of alginate/kaolin beads. The theoretical adsorption capacity (q_max_) did not significantly differ when using pseudo-first-order and pseudo-second-order models. The q_max_ was 0.54 ± 0.040 mg g^−1^ for the alginate/montmorillonite beads and 0.341 ± 0.023 mg g^−1^ for the alginate/kaolin beads. The experimental adsorption capacity of the alginate/montmorillonite and alginate/kaolin beads was 0.398 mg g^−1^ and 0.202 mg g^−1^, respectively. The adsorption kinetic parameters obtained by the pseudo-second-order model best provided a correlation with the experimental data. A similar heterogeneous process was reported for the removal of aqueous Ba^2+^ by natural and Fe(III) oxide-modified allophane, beidellite, and zeolite adsorbents [44]. The pseudo-second-order kinetic model best described the removal of Cu(II) and Pb(II) from wastewater in the case of Mxene/alginate composites [47], and of Pb(II) from an aqueous solution with activated carbon produced from palm fiber with treated oil [48].

Langmuir and Freundlich adsorption models were used to obtain the adsorption isotherms. The isotherms were obtained at 3 mg L^−1^ copper ion concentration. The Langmuir adsorption isotherm quantitatively describes the formation of a monolayer of adsorbate on the outer surface of the adsorbent, after which, no further adsorption takes place.

The parameters of the Langmuir and Freundlich adsorption isotherms are reported in Table 4.

The equilibrium adsorption process was favorable for both adsorbents, because the dimensionless separation factor (R_L_) was between 0 and 1. For the alginate/montmorillonite and alginate/kaolin beads, the maximum monolayer adsorption capacity (q_max_) was close to the experimental value (*q_e_*) for Cu(II) removal, indicating values of 0.6802 ± 0.02 mg g^−1^ and 0.3389 ± 0.03 mg g^−1^, respectively. The Langmuir model showed higher R^2^ than that the Freundlich model for all prepared alginate/clay beads, indicating the monolayer adsorption capacity of the alginate/clay hybrid composite materials. All alginate/clay beads showed a value of inverse adsorption intensity of the process (1/n) below 1, which indicates large Cu(II) adsorption. The K_F_ values for alginate/montmorillonite and alginate/kaolin beads were higher (2.864 and 7.433, respectively), indicating a great removal capacity for Cu(II) as a pollutant from synthetic waters. The phenomenon is in accordance with other studies regarding heavy metals adsorption on different activated adsorbents [49,50,51,52].

**Figure 11:**
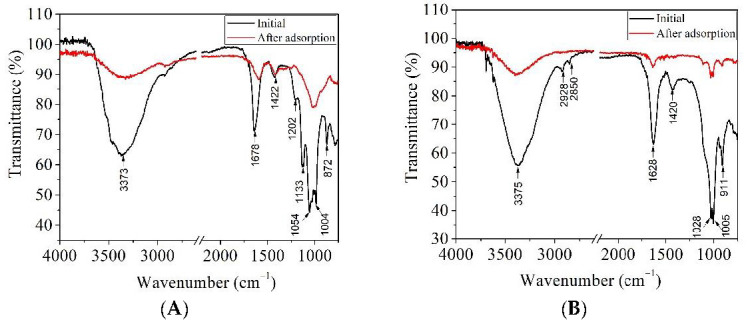
FT-IR spectra for alginate/montmorillonite beads (**A**) and alginate/kaolin beads (**B**), before and after the adsorption process.

In order to evidentiate the interactions between Cu(II) retained on the alginate/clay hybrid beads, FTIR analysis was performed (Figure 11).

As it is observed in Figure 11, two significant bands located in the range of 3373–3375 cm^−1^ (-OH) and 2928–2850 cm^−1^ (C-H groups) decreased in intensity, indicating the formation of hydrogen bonds between the pollutant and the hydroxyl groups of alginate and clay. Similar chemical interactions were found in the case of a cellulose/clay composite hydrogel developed for the adsorption of dye [22].

## 4. Conclusions

Alginate/montmorillonite and alginate/kaolin beads were prepared by the ionic gelation method. Activated clays showed improved surface properties, nanometric size, and polydispersity, and were able to retain heavy metal pollutants. The SEM and FTIR results highlighted the interactions between the alginate and the clays, leading to the formation of hybrid composite beads with better adsorption properties for Cu(II) than the original clays.

The optimal contact time for Cu(II) removal from an aqueous solution was 210 min for the alginate/montmorillonite beads and 135 min for the alginate/kaolin beads. The adsorption processes followed a pseudo-second-order kinetic for the alginate/montmorillonite beads and an intraparticle diffusion model for the alginate/kaolin beads. The alginate/montmorillonite and alginate/kaolin beads well fitted the Langmuir isotherm model and exhibited an adsorption capacity between 0.33 and 0.68 mg g^−1^ for Cu(II) removal. This study demonstrates that the low-cost hybrid material beads based on alginate, montmorillonite, and kaolin can be recommended as promising adsorbents for Cu(II) removal from wastewaters, with improved adsorption properties.

## Figures and Tables

**Table 1:** Elemental composition (weight %) of untreated montmorillonite, treated montmorillonite, untreated kaolin, and treated kaolin.

Element	Untreated Montmorillonite	Untreated Kaolin	Treated Montmorillonite	Treated Kaolin
Carbon (C)	12.45	N.D.	N.D.	59.42
Oxygen (O)	48.84	60.15	52.62	N.D.
Natrium (Na)	N.D.	N.D.	1.19	0.40
Magnesium (Mg)	1.53	0.44	1.60	0.53
Aluminum (Al)	8.08	15.83	10.20	18.57
Silicon (Si)	16.05	21.81	27.89	19.96
Phosphorus (P)	N.D.	0.03	N.D.	0.04
Potassium (K)	2.60	1.03	2.36	0.46
Calcium (Ca)	4.61	N.D.	N.D.	N.D.
Titanium (Ti)	0.66	0.17	0.52	0.14
Iron (Fe)	4.29	0.55	3.64	0.48

N.D., nondetectable.

**Table 2:** Particle size and polydispersity index of the treated clays.

Clay	Z-Average (nm)	PdI	Size for Peak 1 (nm)	Size for Peak 2 (nm)
Treated montmorillonite	899.5 ± 4.738	0.697 ± 0.33	515.1 ± 104	71.15 ± 20
Treated kaolin	667 ± 13.08	0.614 ± 0.054	369.0 ± 33.8	-

**Table 3:** Kinetic parameters obtained for alginate/clay beads according to the three pseudo-kinetic models.

Adsorbent	Pseudo-First-Order Kinetic Model			Pseudo-Second-Order Kinetic Model			Intraparticle Diffusion Model		
	K_1_ (L/min)	q_max_ (mg g^−1^)	R^2^	K_2_ (g/mg min)	q_max_ (mg g^−1^)	R^2^	K_i_ (mg/g min^0.5^)	Ci	R^2^
Alginate/Montmorillonite	0.0175	0.440 ± 0.030	0.9892	0.0253	0.540 ± 0.040	0.99435	0.027 ± 0.0014	0.008 ± 0.016	0.95709
Alginate/Kaolin	0.0230 ± 0.0011	0.270 ± 0.080	0.9268	0.0308	0.341 ± 0.023	0.95532	0.016 ± 0.0006	0.02 ± 0.005	0.98567

**Table 4:** Parameters of the Langmuir and Freundlich adsorption isotherms.

Adsorbent	Langmuir Parameters				Freundlich Parameters		
	q_max_ (mg g^−1^)	K_L_	R_L_	R^2^	K_F_	1/n	R^2^
Alginate/Montmorillonite	0.6802 ± 0.02	1.225	0.7655	0.9799	2.864	0.505	0.8593
Alginate/Kaolin	0.3389 ± 0.03	0.665	0.8572	0.9400	7.433	0.472	0.8426

## Data Availability

The data presented in this study are available on request from the corresponding author.
